# Diagnostic accuracy of Xpert MTB/RIF assay and non-molecular methods for the diagnosis of tuberculosis lymphadenitis

**DOI:** 10.1371/journal.pone.0222402

**Published:** 2019-09-16

**Authors:** Mengistu Fantahun, Abebaw Kebede, Bazezew Yenew, Tufa Gemechu, Yeshiwondm Mamuye, Mengistu Tadesse, Bereket Brhane, Aisha Jibriel, Dawit Solomon, Zelalem Yaregal

**Affiliations:** 1 Pathology, St. Paul’s Hospital Millennium Medical College, Addis Ababa, Ethiopia; 2 Department of Microbiology, St. Paul’s Hospital Millennium Medical College, Addis Ababa, Ethiopia; 3 Ethiopian Public Health Institution, Addis Ababa Ethiopia; 4 Pathology, Addis Ababa University, Addis Ababa, Ethiopia; Central University of Tamil Nadu, INDIA

## Abstract

**Background:**

Tuberculous lymphadenitis (TBLN) diagnosis remains a challenge in resource limited countries like Ethiopia. Most diagnostic centers in Ethiopia use smear microscopy, but it has low sensitivity in detecting tubercle bacilli in fine needle aspiration (FNA) specimens. FNA cytology (FNAC) is another widely applicable diagnostic option but it has low specificity for diagnosing TBLN. In 2014, WHO recommended Xpert MTB/RIF assay to be used in detecting TB from FNA specimen by considering the diagnostic limitations of microscopy and cytology. In Ethiopia, there is limited data on Xpert MTB/RIF performance in detecting TBLN from FNA. Therefore, this study aimed to evaluate the diagnostic performance of Xpert MTB/RIF assay and non-molecular methods (cytology, microscopy and culture) for the diagnosis of TBLN.

**Methods:**

A cross-sectional study was conducted on 152 presumptive TBLN patients at St. Paul’s Hospital Millennium Medical College (SPHMMC) from December 2015 to May 2016 in Addis Ababa, Ethiopia. FNA specimens were collected from each patient. Individual patient specimens were examined by microscopy (acid fast and auramine O staining), cytology, Xpert MTB/RIF and culture. Each specimen was directly inoculated and its sediment following decontamination procedure onto two duplicate Löwenstein-Jensen (LJ) media. Composite culture (specimen positive by direct or concentrated or both culturing methods) and composite method (positive by either one of the non-molecular methods) were taken as reference methods. The data was captured and analyzed using software packages SPSS version 20 (SPSS Inc, Chicago, Illinois, USA). Sensitivity, specificity, positive predictive value, and negative predictive value were calculated.

**Result:**

A total of 152 presumptive TBLN patients were enrolled in this study. Of these, 105(69%), 68(44.7%), 64(42%), 48(32%) and 33(22%) were positive for *M*. *tuberculosis* using composite method (positive by either one of the non-molecular method), composite culture, direct, and concentrated culture, respectively. TB positivity rate was 67.8%, 49.3%, 24.3%, and 14.5% using cytology, Xpert MTB/RIF, Auramine O (FM) microscopy, and Ziehl Nelson (ZN) microscopy, respectively. Using composite culture as reference, the sensitivity and specificity of Xpert MTB/RIF was 78% (95% CI: 73.7% to 82.3%) and 74% (95%CI: 69.4% to 78.6%), respectively. However, the sensitivity of Xpert MTB/RF improved from 78% to 92% using composite method as a reference. The high positivity rate observed in purulent (70%) followed by caseous (66.7%) type of aspirates by Xpert MTB/RIF.

**Conclusion:**

Xpert MTB/RIF assay has both considerable sensitivity and specificity; it may be employed for better diagnosis, management and treatment of presumptive TBLN patients.

## Introduction

Globally, Ethiopia ranks 7^th^ among the 22 high burden TB countries (HBTCs) and second in terms of extrapulmonary TB (EPTB) cases [1]. EPTB accounts for 36% of new TB cases [2]. Among EPTB, TBLN represents majority cases [3]. The prevalence of culture confirmed TBLN is also reported above 50% [3, 4].

To reach the first milestone of the End TB strategy, TB annual decline rate should be accelerating 4–5% by 2020. Since significant diagnostic gaps persisting; globally, TB annual decline rate remained at only 1.5% [5].

Detecting TBLN from FNA sample is challenging in Ethiopia because it uses mostly conventional methods [6]. Ziehl Nelson (ZN) smear microscopy has low sensitivity in the detection of tubercle bacilli in FNA specimens [7,8]. However, light emitting diode (iLED) based fluorescence smear microscopy 10 times more sensitive than the ZN microscopy [9]. It may also improve the detection of TB in paucibacillary EPTB specimens like FNA since it allows examination of more fields with less effort [10,11].

Cytology is the most routinely utilizing diagnostic tool for the diagnosis of TBLN. In TB prevalent area like Ethiopia; TB diagnosis can be made confidently when it’s cytomorphological features are met [12]. The cytological criteria for diagnosis of possible TBLN have been clearly defined as epitheloid cell granulomas with or without multinucleated giant cells and caseation necrosis [13]. The cytological diagnosis has low specificity [3]; however, its specificity reported as high as 88% [14].

Mycobacterial culture is a technique considered to be a gold standard for TB laboratory diagnosis. However, the technique is not widely practiced in resource limited countries because of a minimum requirement of biosafety level II TB laboratory [15]. The culture technique has also long turnaround time (TAT) due to the slow growing nature of *M*. *tuberculosis* (MTB). TAT can be affected by the bacilli load in clinical specimens. Additionally, the paucibacillary nature of FNA specimens could affect the TAT [15,16]. In line with these limitations more rapid and reliable methods are needed.

PCR based molecular assays thought to be more sensitive than microbiological tests and have better specificity than the cytology. Moreover, PCR technologies have short TAT, mostly a few hours, since mycobacterial DNA materials are the target for detection of TB.

A rapid molecular assay like Xpert MTB/RIF with 2 hours TAT benefits the patient and the community by early detection and initiation of treatment. In some studies, the sensitivity of Xpert MTB/RIF much better than the mycobacterial culture technique and suggested being used as a reference method [17]. The Xpert MTB/RIF assay consists of a closed system that is based on real-time polymerase chain reaction (PCR). It can be used by operators with minimal technical expertise, enabling the diagnosis of TB and simultaneous detection of rifampicin resistance within 2 hours [18].

The Xpert assay has been validated and optimized for sputum samples to diagnose HIV-associated TB and rifampicin-resistant TB. WHO strongly recommends widespread use of Xpert for these groups of patients [19,20]. More recently a number of studies were done to evaluate this assay using non-respiratory clinical samples from patients presumed of having EPTB [21,22,23]. In 2014, WHO has recommended Xpert over the conventional tests (including conventional microscopy, culture or histopathology) for testing specific non-respiratory specimens (lymph nodes and other tissues) from patients presumed of having EPTB [24]. However, this was a conditional recommendation due to very low-quality evidence available.

More studies are therefore needed particularly in settings with high EPTB prevalence. Therefore, this study aimed to evaluate the diagnostic performance of Xpert MTB/RIF assay in comparing against the non-molecular conventional methods (cytology, microscopy, and culture) for the diagnosis of TBLN.

## Materials and methods

### Study setting and patient recruitment

A cross-sectional study was conducted at SPHMMC, Addis Ababa, Ethiopia. One hundred fifty two clinically presumptive TBLN patients who visited the Pathology Department of SPHMMC were enrolled in this study during the period between December 2015 and May 2016.

Informed consent and assent were obtained from the study participants and guardian, respectively. Demographic and clinical data for all patients were collected using a pretested structured questionnaire by trained medical laboratory scientists. Patients who were taking anti-TB treatment at the time of sample collection and who had inadequate sample were excluded from the study. The result of the patients was reported to the respective physicians for treatment. Information obtained at any course of the study was kept confidential. In addition, the collected clinical specimen was used only for the stated objectives of the study.

### Sample collection and processing

FNA was collected from each presumed to be TBLN patients under aseptic condition. Clinical features were provided by clinicians and confirmed by pathologists. The collected FNA transferred from the5ml syringe into sterile 50ml Falcon tube, and three smears were made directly from the syringe on-site. The transferred FNA specimens were transported to National TB Reference Laboratory (NTRL) of Ethiopian Public Health Institute (EPHI) using a cold chain system. The FNA specimen divided equally (Sample 1 and Sample 2) using another sterile 50ml Falcon tube inside the biosafety cabinet at NTRL, EPHI. One half FNA specimens (Sample 1) were tested by Xpert MTB/RIF assay. Few drops from Sample 2 directly inoculated into two LJ media. The remaining portion of Sample 2 was decontaminated and concentrated by N-acetyl-L-cysteine (NALC)/sodium hydroxide (NaOH)-Sodium (Na) Citrate method [25,26] and then inoculated into another LJ medium.

Two of the three smears were stained with acid-fast and Auramine O stains for ZN and fluorescence microscopic examination, respectively. The remaining smear was stained with Wright stain at Pathology laboratory for cytology examination.

## Operational definition

**Composite culture** refers to culture result by combining the direct LJ culture and concentrated LJ culture.

**Positive composite culture** is defined as any FNA specimen positive to either of the two cultures inoculates (direct or concentrated) or both cultures inoculate (direct and concentrated).

**Composite method** refers to MTB positive by non-molecular method (cytology, Auramine O (FM) microscopy and Ziehl Nelson (ZN) microscopy).

**Bacteriological confirmed TB case** refers to TB positive specimen (patient) in any of the bacteriological diagnostic technologies; microscopy, Xpert MTB/RIF assay and composite culture.

## Laboratory methods

### Xpert MTB/RIF assay

An equal volume of sample reagent was added into Sample 1 (1:1 ratio). Mixed well twice during the 15 minutes incubation time at room temperature. Two milliliters of the mixture was transferred in to test cartridge. The test cartridge was loaded into the Gene Xpert machine. *M*. *tuberculosis* Complex (MTBC) positivity and its rifampicin resistance were determined within 2 hours [16].

### Cytological diagnosis

One air dried FNA smear from an individual patient was stained with Wright stain as described elsewhere [27] and examined microscopically by experienced pathologists. FNA smear would be considered as TB positive if cytology showed epithelioid granuloma with or without multinucleated giant cells and with or without caseous necrosis and/or liquefied necrotic material with degenerating and viable inflammatory cells without epitheloid granuloma [12].

### Ziehl Nelson (ZN) smears microscopy

The second air dried FNA smear was stained with ZN staining procedure [28]; the stained smear examined under the oil immersion (100X) objective of a light microscope (Olympus C × 31- Japan); a minimum of 100 fields of the stained smear should be scanned to declare as negative [8].

### Light emitting diode (LED) based fluorescence smear microscopy (FM)

Direct FNAs smeared on one slide and air dried and then stained with Auramine O staining procedure [26] and examined using iLED fluorescence microscopy (FM) (ZEISS primo star) under low power (40X) objective; with no darkroom requirement. The bacilli appear as slender bright yellow fluorescent rods; standing out clearly against a dark background. At least 30 fields of the smear should be scanned to declare as negative [10].

### Mycobacterial culture

Few drops from Sample 2 directly inoculated onto two Lowenstein–Jensen (LJ) slants and incubated at 37°C. The remaining portion of Sample 2 was digested, decontaminated and concentrated by N-Acetyl-L-Cysteine/Sodium Hydroxide-Sodium Citrate (NALC/NaOH-Na Citrate) decontamination procedure [27]. Thereafter, the specimen was concentrated by centrifugation at 3500g for 15 minutes and resuspended in 1ml of sterile phosphate buffer (pH = 6.8). The suspended sediment was inoculated onto two Lowenstein–Jensen (LJ) medium slants and incubated at 37°C. Both the direct and concentrated culture was examined on a weekly basis for mycobacterial growth for eight consecutive weeks [18].

### Statistical analysis

The data was captured and analyzed using software packages SPSS version 20 (SPSS Inc, Chicago, Illinois, USA). Sensitivity and specificity, positive predictive value and negative predictive value were calculated for assessing the performance of Xpert MTB/RIF, microscopy and cytology against the composite culture result. *P<0*.*05* was considered as statistically significant.

## Result

### Demographic and clinical data

In this study, 152 clinically presumed to be TBLN patients were enrolled as per the inclusion criteria. The median age of the patients was 27 (range 5–72) years. The majority of the patients were female (55.9%), primary school (50.7%), married (41.4%) and daily laborer (30.9%). Most of the patients had no previous history of treatment. The prevalent diabetes mellitus was 10.5%. The hemorrhagic aspirate was the predominant type among the collected FNA specimens (Table 1).

**Table 1:** Demographic and clinical characteristics of presumptive TBLN patients.

Socio-demographics	Categories	n(%)
Sex	Male	67(44.1)
	Female	85(55.9)
Age (Years)	0–14	18(11.8)
	15–24	44(28.9)
	25–34	41(27.0)
	35–44	24(15.8)
	45–54	14(9.2)
	>54	11(7.2)
Educational status	Illiterate	42(27.6)
	Primary school	77(50.7)
	Secondary school	25(16.4)
	Higher education	8(5.3)
Occupation	Housewife	13(8.6)
	Daily laborer	47(30.9)
	Government employee	12(7.9)
	Unemployed	38(25.0)
	Farmer	36(23.7)
	Prisoner	2(1.3)
	Other	4(2.6)
Residency	Urban	84(55.3)
	Rural	68(44.7)
Marital status	Living with a partner	14(9.2)
	Married	63 (41.4)
	Divorced	6 (3.9)
	Single	63 (41.4)
	Widowed	4 (2.6)
	Separated	2 (1.3)
Aspirate types	Hemorrhagic	60 (39.5)
	Pus	50 (32.9)
	Caseous	42 (27.6)
Previous TB treatment	New	127 (83.5)
	Previously treated	25(16.5)
Diabetes	Yes	16 (10.5)
	No	136 (89.5)

### TB positivity rate among presumptive TBLN patents using different TB diagnostic tools

One hundred fifty-two FNA specimens were collected at the Pathology Department of SPHMMC. Xpert MTB/RIF indicated that 49.3% of the presumed to be TBLN patients had TB lymphadenitis. TB positivity rate was 67.8%, 44.7%, 24.3%, and 14.5% by using cytology, LJ culture, FM, and ZN, respectively (Table 2).

**Table 2:** TBLN positivity by different diagnostic methods (N = 152).

Methods	Positive	Negative
Cytology, n (%)	103(67.8%)	49(32.2%)
Xpert MTB/RIF assay, n (%)	75(49.3%)	77(50.7%)
Composite culture, n (%)	68(44.7%)	84(55.3%)
FM, n (%)	37(24.3%)	115(75.7%)
ZN, n (%)	22(14.5%)	130(85.5%)

### Performance of Xpert MTB/RIF and non-molecular tests for detection of TBLN

The diagnostic performance of Xpert MTB/RIF, cytology and microscopy were evaluated against the reference composite culture result. The sensitivity was 94.1%, 78%, 47%,and 30.9%, for cytology, Xpert MTB/RIF, FM and ZN, respectively. However, the specificity was 98.8%, 94%, 74% and 53.6% cytology, Xpert MTB/RIF, FM, and ZN, respectively. Xpert MTB/RIF has reasonable sensitivity (78%) and specificity (74%)(Table 3).

**Table 3:** Performance of Xpert MTB/RIF and non-molecular methods against composite culture for the diagnosis of presumptive TBLN patients.

Laboratory methods	Sensitivity (95%CI)	Specificity (95%CI)	PPV (95%CI)	NPV (95%CI)
Cytology	94.1%(91.5–96.5)	53.6%(48.4–58.8)	62.1%(57.1–67.1)	91.8%(89–94.6)
Xpert MTB/RIF	78%(73.7–82.3)	74%(69.4–78.6)	71%(66.3–75.7)	81%(76.9–85.1)
ZN	30.9%(26–35.7)	98.8%(97.8–99.8)	95.5%(93.5–97.5)	63.8%(58.8–68.8)
FM	47%(941.8–52.2)	94%(91.5–96.5)	86.5%(83–90)	68.7%(63.9–73.5)

The sensitivity and specificity of Xpert MTB/RIF improved from 78% to 92%, 74% to 98.7% using composite method (positive by either one of the non-molecular method) as a reference (Table 4).

**Table 4:** Performance of Xpert MTB/RIF against composite method for the diagnosis of presumptive TBLN patients.

Operational characteristics	Xpert MTB/RIF
Sensitivity (95%)	92% (91.8–92.5)
Specificity (95%)	98.7% (95.6–98.2)
PPV (95%)	96.9% (94.1–96.3)
NPV (95%)	89.6% (82.9–88.1)

### Aspirate types and detection of TBLN using Xpert MTB/RIF and non-molecular tests

The majority of the aspirates were hemorrhagic (39.5%). Xpert MTB/RIF showed the highest detection rate (70%) of caseous aspirate, but the low number of TBLN (20%) cases were identified from the hemorrhagic type of aspirate.

*M*. *tuberculosis* was detected in 38.3%, 15%, 3.3% and 1.7% of hemorrhagic aspirates by cytology, culture, FMand ZN, respectively.

Cytology revealed TBLN in 80% of purulent aspirate. But *M*. *tuberculosis* confirmed in 70%, 58%, 40%,and26% by using Xpert MTB/RIF, culture, FM and ZN, respectively. Seventy percent of caseous aspirates were confirmed by Xpert MTB/RIF assay. Therefore, being caseous aspirate highly indicative of TBLN. And aspirate types had statistically significant with confirmed TBLN **(***p<0*.*001*) (Table 5).

**Table 5:** Detection of TBLN in association with aspirate types using Xpert MTB/RIF and non-molecular assays.

	Laboratory methods										
	Xpert M TB/RIF		Cytology		Culture		FM		ZN		Total
Types of aspirate	Pos (%)	Neg n (%)	Pos n (%)	Neg n (%)	Pos n (%)	Neg n (%)	Pos n (%)	Neg n (%)	Pos n (%)	Neg n (%)	
Hemorrhagic	12(20)	48(80))	23(38.3)	37(61.7)	9(15)	47(85)	2(3.3)	58(96.7)	1(1.7)	59(98.3)	60
Purulent	35(70)	15(30)	40(80)	10(20)	29(58)	21(42)	20(40)	30(60)	13(26)	37(74)	50
Caseous	28(66.7)	14(33.3)	40(95.2)	2(4.8)	30(71.4)	12(28.6)	15(35.7)	27(64.3)	8(19)	34(81)	42

*Pos = positive, *Neg = negative

## Discussion

In our study, females were slightly more common than males (55.9% vs. 44.1%). A similar observation was noted by Ergete *et al* (8). This high number of females to be suffering from lymph node swelling could be the females in developing countries have a weak immune system; they always do more work, consume low quality food and bear the high nutritional and physical burden during repeated pregnancies and lactation [29]. The age group of patients with lymph node swelling was younger with 25–34 years old being affected accounting 32% followed by 15–24 years old accounting 29.2% of the cases. This finding is inconsistent with other studies in Ethiopia, where younger than 30 years old are the commonest age group affected by this disease [18,26].

Xpert MTB/RIF assay positivity rate was 49.3%. Ten samples were detected in Xpert MTB/RIF assay but negative in FNAC. This might be due to early stage of the disease, inadequate cellular change to be seen in the light microscope. The sensitivity of Xpert MTB/RIF assay was 78%. This finding is lower than another study from Ethiopia done by Mulualem *et al* (sensitivity, 87.8%) [29]. The specificity of the X pert MTB/RIF assay against composite culture was 74%. This is in agreement with the study done by Biadigilegn *et al* (specificity, 69.2%) [2]. But it improved from74%to 98.7% when the composite method used as a reference.

TBLN diagnosis mostly re1lies on cytology but it is not bacteriological detection and based on cell morphology changes; its specificity is confounded by other inflammatory reactions [26]. Furthermore, it cannot rule out tuberculous and NTM causes [27]. Our finding showed that 68.7% was positive using cytology; which is in concordance with study (69.5%) in Ethiopia by Muluye *et al* [3]. However, another study in southern Ethiopia showed significantly lower prevalence (48.8%) [30]. This difference might be the patients living in a different geographic locations, and the progress of the disease at the time of data collection.

In our study, the sensitivity and specificity of cytology against composite culture was 94.1%, and 53.6%, respectively. This specificity was in agreement with specificity (50%) done by Derese *et al* Ethiopia [16]. When cytology compared against bacteriological MTB result the sensitivity and specificity of the cytology was 87.8% and 61.3%, respectively. There was no statistically significant difference between the specificity of cytology compared against composite culture and bacteriological MTB result. Overall, cytology lacks specificity but has high sensitivity [31,32]. This may be due to confounding by other inflammatory reactions (not caused by tuberculosis). Therefore, believing in cytology alone might lead to false case reporting and consequently result in incorrect patient management and inappropriate use of anti-TB drugs, and finally misleading policy makers.

At least 10 live bacilli found in one ml sample to get a positive result on culture. In our study 44.7% of cases of composite culture proven TBLN patients were identified. This finding is higher than a study done by Garedew *et al* (36.7%) [33]. Our finding was lower than a study done by Olifan *et al* (56.1%) [34]. This lower finding could be due to previous treatment by one or more course anti-biotic that could suppress and inhibit bacterial growth and scanty nature of the bacilli in FNA samples [35, 36].

In our study, high culture positivity rate (71.4%) was recorded in caseous types of aspirates. The reason may be a high number of bacterial load in caseous aspirates with an advanced stage of the disease, this also supported by another study [37].

There is considerable evidence of increased effectiveness of using Auramine O stain to demonstrate AFB as compared to the ZN method. In this study, FM gave higher positivity (24.3%) than ZN (14.5%), hence FM is the most sensitive conventional method but less specific; falsely positive result.

Direct FM has detected 47% of culture-confirmed TBLN cases this is might be due to low bacilli found in the FNA sample. And five cases were positive in FM but culture negative this possibly dead bacilli could not grow on culture. The FM diagnostic specificity was 94% and sensitivity was 47%, against composite LJ culture used as the gold standard. If cytology is supported by ZN stain for AFB detection then it becomes a reliable and valuable diagnosis of TBLN and treatment can be started without any reservation.

In our study, maximum positivity of AFB (59%) was observed in pus aspirates and it is well supported by another study conducted by Nidhi *et al* where they found 85.5% [38]. The reason may be AFB concentration is more in the aspirates showing purulent or necrotic material [39].

Ziehl Neelsen staining to identify the AFB in FNA is a simple and affordable method. Our study showed that 22(14.5%) was diagnosed as TBLN. This finding has low positive rate, as investigating bacilli in a clinical specimen by ZN requires more than 5,000 organisms/ml of sample, but very low bacilli expected in FNA sample; another investigator also agrees with this explanation [40]. But some studies had higher detection rate [32, 41]. The uneven distribution of bacilli in FNA may be making a difference between our finding and their findings. Even though the sensitivity was found to be relatively low (30.9%), the high specificity (98.8%) was observed. Therefore, ZN alone gives the confidence to start anti TB treatment; this is very crucial to eliminate antibiotics and anti-TB trials. The quality of the smear, the method used and the scanty bacilli found in the FNA could be the main factor for decreased sensitivity.

## Conclusion

From this finding, we conclude that the current prevalence of TBLN with the gold standard technique was high (44.7%). Xpert MTB/RIF assay has both considerable sensitivity and specificity than other methods; it may be employed for better diagnosis, management, and treatment of presumptive TBLN patients.

## Abbreviations

EPHI: Ethiopian Public Health Institute
FNA: Fine needle aspirate
MTB: *Mycobacterium tuberculosis*
NTM: Nontuberculous Mycobacteria
SPHMMC: St. Paul’s Hospital Millennium Medical College
TBLN: Tuberculosis lymphadenitis
WHO: World Health Organization
